# Clinical Implications and Patient Outcomes Associated with Antibiotic Resistance in Saudi Arabian Intensive Care Unit Facilities: A Perspective

**DOI:** 10.2174/0127724328391436250902082555

**Published:** 2025-09-03

**Authors:** Saad S. Alqahtani, Muhammad H. Sultan, Sivakumar S. Moni, Renju Ravi, Ali Alshahrani, Santhosh Joseph Menachery

**Affiliations:** 1 Department of Clinical Pharmacy, College of Pharmacy, King Khalid University, Abha, Saudi Arabia;; 2 Department of Pharmaceutics, College of Pharmacy, Jazan University, Jazan, Saudi Arabia;; 3 Health Research Centre, Jazan University, Jazan, Saudi Arabia;; 4 Department of Basic Medical Sciences, Faculty of Medicine, Jazan University, Jazan, Saudi Arabia;; 5 Department of Clinical Pharmacy, College of Pharmacy, Taif University, Taif, Saudi Arabia;; 6 Department of Clinical Practice, College of Pharmacy, Jazan University, Saudi Arabia

**Keywords:** Saudi Arabia, antibiotic resistance, intensive care units, clinical implications, resistance patterns, prevention, challenge

## Abstract

**Introduction:**

Antimicrobial Resistance (AMR) poses a significant global health threat, leading to increased morbidity, mortality, and healthcare costs. Intensive Care Units (ICUs) are particularly susceptible to AMR due to frequent invasive procedures, extended hospital stays, and the selective pressure exerted by broad-spectrum antibiotics. This review aims to shed light on the current landscape of antibiotic resistance within ICUs of Saudi hospitals. It also explores molecular patterns of bacterial resistance and identifies potential strategies to address this issue. Additionally, it discusses the challenges in implementing these strategies within the Saudi healthcare system.

**Methods:**

We conducted a literature search across electronic databases, including Web of Science, PubMed, EMBASE, Scopus, and Google Scholar, until September 30th, 2024, to identify relevant studies. Selected studies were analyzed to extract insights into prevailing bacterial resistance trends in Saudi ICUs and the molecular mechanisms responsible.

**Results:**

Our findings provide an overview of the current state of AMR in Saudi ICUs, including the emergence and prevalence of specific molecular patterns of bacterial resistance. Moreover, it presents potential strategies to combat antibiotic resistance, including antimicrobial stewardship programs, infection control measures, and the development of new antibiotics. It also highlights the inherent challenges in implementing these strategies within the unique healthcare landscape of Saudi Arabia.

**Discussion:**

The increasing emergence and spread of MDR bacteria in Saudi Arabia are attributed to the unoptimized antibiotic use, over-the-counter antibiotics without prescription, a high volume of international travellers, and challenges in adherence to infection control practices. Addressing the challenges and implementing effective prevention strategies are critical to maintaining antibiotic efficacy and combating AMR. Several strategies have been employed by the National AMR Committee, in partnership with WHO, to address antibiotic resistance in intensive care units.

**Conclusion:**

AMR in Saudi ICUs is a pressing concern requiring immediate attention. A multifaceted approach combining surveillance, education, and policy interventions is essential to overcome this issue. Addressing AMR is crucial for global efforts to preserve the efficacy of antibiotics and maintain the effectiveness of critical healthcare interventions.

## 1 INTRODUCTION

The overuse and misuse of antibacterial agents are the main cause of the widespread increase in antibiotic resistance [1]. The World Health Organization (WHO) defines Antimicrobial Resistance (AMR) as a natural phenomenon that occurs when microorganisms stop responding to antimicrobials. AMR results in drug resistance, which in turn makes infections harder to treat and increases the risk of disease spread, disability, and death. In 2019, bacterial AMR alone was responsible for 1.27 million deaths and contributed to 4.95 million deaths worldwide [2]. In Saudi Arabia, about 2,500 deaths were attributed to AMR [3]. The neighboring countries, such as Iran, had higher deaths (6,900) due to AMR [4], followed by Iraq with 3,400 deaths [5]. In contrast, Oman (177 deaths) [6], Kuwait (197 deaths) [7], and UAE (418 deaths) [8] had relatively lower mortality rates. Globally, bacterial resistance has become a therapeutic challenge, especially for Hospital-Acquired Infections (HAIs), as hospitalized patients are more susceptible to infection for various reasons. In addition, HAIs are caused by a variety of bacteria, many of which have developed Multiple Antibiotic Resistance (MAR) as a result of extensive antibiotic use in hospitals. The emergence and spread of hospital-acquired MAR bacteria is a major public health concern because these bacteria can cause infections that are difficult to treat. Researchers have used various methods, including genome sequencing, transcriptomics, and proteomics, to understand the molecular patterns of MAR bacteria. The WHO estimates that 7 million people die annually from healthcare-associated diseases [1]. According to a 2021 study published in the Journal of Hospital Infection, up to 50% of Intensive Care Unit (ICU) patients receive antibiotics, with 30% receiving them inappropriately [9].

Socioeconomic factors such as income level play a role in antibiotic misuse, including nonadherence and unprescribed use. According to Mallah *et al.*, the likelihood of antibiotic misuse is similar among low- and high-income individuals, which aligns with the fact that both groups tend to self-medicate. People with low incomes self-medicate to avoid expenses related to consultation and lab tests, while those with high incomes self-medicate as they have easier access to health information on the internet, can afford to purchase non-reimbursed medicines, and benefit from greater social support, increasing their access to unprescribed medicines [10]. In addition to the misuse and overuse of antibiotics, other risk factors for HAIs in ICUs include invasive medical procedures, contaminated medical equipment, and poor hand hygiene among healthcare workers [11].

Apart from socioeconomic factors, cultural practices, public misconceptions, and media influence are also responsible for self-medication. A nationwide survey in Saudi Arabia by Alhur *et al.* revealed that 59.8% of participants believed it is safe to self-medicate, and 75.5% of the participants engaged in self-medication [12]. A cross-sectional study in Qassim province, Saudi Arabia, showed that there is a high prevalence of non-prescribed medicine purchases, and the most commonly purchased drugs include antibiotics, analgesics, and antiallergics [13]. Another study reported that the majority of Saudi Arabs engage in self-medication and seek advice from friends and family rather than consulting a doctor. The lack of medical insurance, not having time to visit the doctor, and reluctance to go to the hospital are some of the reasons for self-medication [14].

The consequences of healthcare-associated infections in ICUs can be severe and even life-threatening, particularly for critically ill patients. Healthcare-associated infections lead to longer hospital stays, higher costs, and increased mortality rates, and can also contribute to the development of antibiotic-resistant bacteria, making treatment more challenging. As patients infected with antibiotic-resistant microorganisms seldom recover with the first antimicrobial treatment, additional investigations and treatments are necessary. This can result in higher expenses for both hospitals and society. Therefore, preventing and controlling healthcare-associated infections in ICUs is crucial to ensuring the safety and well-being of patients. Strategies such as proper hand hygiene, appropriate use of antibiotics, and environmental cleaning can help reduce the incidence of healthcare-associated infections in ICUs [15-19].

Several initiatives and guidelines have been developed to address the issue of HAIs in healthcare settings. The Centers for Disease Control and Prevention’s guidelines and the WHO’s global campaign, called the Global Patient Safety Challenge: Cleaner Care, are examples [20, 21]. In a previous study published in the Saudi Medical Journal, the incidence rate of HAIs in ICUs in Saudi Arabia was 20.2%. This is significantly higher than the global average of 8.7m% [22, 23]. The high prevalence of HAIs in Saudi Arabian hospitals can be attributed to various factors, including inadequate infection control measures, overcrowding, insufficient resources, and poor staff compliance with hygiene protocols. Additionally, the overuse and misuse of antibiotics in Saudi Arabia have contributed to the emergence of antibiotic-resistant bacteria, which further exacerbates the problem of HAIs [24, 25]. In order to resolve the issue, the Saudi Ministry of Health (MOH) has implemented several initiatives to improve infection control and prevention measures in hospitals [26]. This includes establishing infection control committees, implementing strict hand hygiene protocols, and developing guidelines for antibiotic use. Several studies have also been conducted in Saudi Arabia to explore the prevalence and risk factors associated with HAIs in ICUs. For instance, a study published in the International Journal of Health Sciences found that the most common types of HAIs in ICUs were ventilator-associated pneumonia and bloodstream infections.

Despite these initiatives, the prevalence of HAIs in Saudi ICUs remains a concern due to factors such as MAR, antibiotic misuse, and the spread of treatment-resistant microorganisms. This review aimed to provide a qualitative understanding of the clinical impact and outcomes associated with antibiotic resistance in Saudi ICUs by focusing on the molecular patterns behind antibiotic resistance, the prevention strategies, and the associated challenges to address this issue. Given the heterogeneous nature of the studies included, which contained data on various resistant microorganisms, a quantitative analysis was not feasible.

## 2 MATERIALS AND METHODS

This document was prepared by conducting a rigorous literature search across electronic databases (Web of Science, PubMed, EMBASE, Scopus, and Google Scholar) using the keywords “antibiotic resistance,” “Saudi Arabia,” and “intensive care units” until September 30, 2024, to identify relevant studies. The studies were analyzed, and the extracted data were interpreted in the following sections: bacterial resistance, molecular patterns of bacterial resistance, strategies of the WHO and Saudi Arabia regarding antibiotic resistance in ICUs, prevention of AMR, and its challenges.

## 3 BACTERIAL RESISTANCE

AMR in hospitals is a major problem worldwide, including in Saudi Arabia. Microorganisms, including microbes and parasites, can resist antimicrobial drugs, making diseases caused by these organisms challenging or difficult to treat. Several factors have exacerbated the problem, including antibiotic misuse, inadequate infection control methods, and a lack of knowledge and education among healthcare professionals and the public. The WHO has also acknowledged that a significant gap exists in knowledge and awareness of AMR among healthcare workers. To overcome this, the WHO has developed a competency framework for the education and training of health workers on AMR [19]. Table **1** shows that HAIs are a significant problem in Saudi Arabia, especially in ICUs [26-32].

In Saudi Arabia, the prevalence of methicillin-resistant *Staphylococcus aureus* (MRSA), *Escherichia coli*, and *Enterobacteriaceae* varies significantly during the Hajj pilgrimage. A study conducted over two Hajj seasons investigated the knowledge, attitude, and practices of healthcare personnel deployed during the pilgrimage. The results revealed that only 19% of the respondents were familiar with antimicrobial stewardship programs, while a significant 85% were aware of AMR [33, 34]. These findings underscore the crucial need to educate and train young physicians about mass gatherings to raise awareness of the potential medical risks and challenges associated with such events.

AMR is linked to higher morbidity and mortality rates, longer hospital stays, higher healthcare costs, and limited treatment options for patients. Treating infections caused by multidrug-resistant organisms like MRSA, *etc*., can be challenging. In addition, these microbes are often associated with serious infections that require aggressive medical treatment, such as ventilator-associated pneumonia, bloodstream infections, and surgical site infections [35]. The Saudi Arabian MOH has implemented infection control measures in hospitals and healthcare facilities to reduce the spread of resistant organisms and promote the prudent use of antibiotics through policies and educational initiatives for medical personnel. There are different types of bacterial resistance prevailing in Saudi Arabia [36]. In the ICU, bacterial resistance can be a major challenge due to the high prevalence of critically ill patients, frequent use of invasive procedures, and prolonged exposure to antibiotics. Various types of bacterial resistance have been identified in ICUs in Saudi Arabia (Fig. **1**) [37-46].

### 3.1 Methicillin-Resistant Staphylococcus aureus (MRSA)

MRSA, a strain of *Staphylococcus aureus* that has developed resistance to methicillin and related beta-lactam antibiotics, renders these drug classes ineffective against infections caused by it. MRSA infections, particularly in ICUs, pose significant challenges to treatment and are linked to higher mortality rates in the general population. Skin and soft tissue infections caused by MRSA often co-occur with infections caused by other bacteria, such as methicillin-resistant *Pseudomonas*.

In Saudi Arabia, a study examined the factors associated with the incidence of MRSA infections and their association with hospitalization, mortality, and the timing of infection [47]. The study analyzed 44 MRSA cases, including patients admitted to the ICU at King Fahd Specialist Hospital in Al-Qassim, Saudi Arabia, between 2015 and 2019, revealing that 73% of cases occurred within the first two weeks of admission. A 10-year surveillance study (2006-2015) from Qatif, Saudi Arabia, found a 13% incidence of MRSA infections in ICUs, similar in age and severity in both adult and pediatric patients, suggesting that the epidemiology of MRSA is age-independent [48]. A retrospective cohort study (January 2013- June 2017) at King Abdulaziz Medical City in Riyadh found a 20.1% 30-day mortality rate for patients with MRSA bacteremia [49]. Most studies on MRSA in Saudi Arabia have focused on epidemiologic patterns, including prevalence and incidence among patients and healthcare workers (*e.g*., nosocomial or community-acquired), various strains, and antibiotic susceptibility.

### 3.2 Extended-Spectrum Beta-Lactamase (ESBL)

ESBL-producing bacteria, such as *Escherichia coli* and *Klebsiella pneumoniae,* are common in ICUs. These bacteria can cause urinary tract infections, bloodstream infections, and other serious infections. Their resistance to several antibiotics, including beta-lactams, limits treatment options. ESBL-producing strains, particularly in *Enterobacteriaceae*, are increasing in Saudi Arabia, with Carbapenem resistance rising, although data on ESBL epidemiology are limited. ESBL-producing isolates from various sample types, including wounds, urine, blood, and aspirates, collected from hospitals in Saudi Arabia, are mainly represented by *Klebsiella pneumoniae* and *Escherichia coli*. The isolates exhibit enhanced resistance to penicillin, extended-spectrum cephalosporins, aminoglycosides like gentamicin, fluoroquinolones like ciprofloxacin, and trimethoprim /sulfamethoxazole. The susceptibility of ESBL-expressing organisms to the carbapenems, including imipenem, meropenem, and ertapenem, was generally high. However, there were significant levels of uncertainty among healthcare workers concerning hospital policies related to reporting or prescribing for these infections. The study highlights the significance of prioritizing staff training and improving communication procedures for infection prevention and control concerning ESBLs [50].

A study conducted at King Abdullah Hospital, Bisha Province, Saudi Arabia, found that 27% of 311 Gram-Negative Bacilli (GNB) in ICU isolates had ESBL, with *Klebsiella pneumoniae* being the most common ESBL producer, followed by *Escherichia coli* [51]. A cross-sectional study conducted at Turaif General Hospital in Saudi Arabia found that *Escherichia coli* was the most common ESBL producer, at 51.4%, followed by *Klebsiella pneumoniae* at 27% [52]. A recent study conducted at King Faisal Hospital in Makkah, Saudi Arabia, found that 20.6% of samples from ICU patients were ESBL-producing GNB, with estimates of ESBL-producing *Escherichia coli*, *Klebsiella pneumoniae*, and other GNB at 44.4%, 41.6%, and 14.0%, respectively, with imipenem being the most effective antibiotic against these bacteria [53].

### 3.3 Carbapenem-Resistant Enterobacteriaceae (CRE)

Carbapenem-resistant bacterial strains such as *Klebsiella pneumoniae* and *Escherichia coli* can lead to severe infections that are difficult to treat because of limited antibiotic options. The occurrence and spread of CRE in healthcare settings, especially intensive care units, is a major problem worldwide, including Saudi Arabia. Most CRE reports originate from Riyadh, Saudi Arabia, while few studies on Enterobacteriaceae susceptibility and molecular characteristics are reported from other regions of the kingdom.

A multicenter prospective cohort study was conducted over 28 months in several Saudi Arabian centers to describe the molecular epidemiology and progression of CRE [54]. The study involved 189 patients, with nosocomial pneumonia (23.8%) and complicated urinary tract infection (23.8%) being the most common CRE infections, with bacteremia affecting 40.7% of the patients. The study found that CRE infections are associated with poor outcomes due to limited treatment options and inadequate initial therapy. Another study from Dammam, Saudi Arabia, demonstrated a low prevalence of gastrointestinal tract colonization of CRE in ICU patients, with a prevalence of 0.5% [55]. A study from King Fahad Medical City, Riyadh, revealed that *Klebsiella pneumoniae (47%)* and *Escherichia coli (31%*) are common causes of CRE infection in pediatric patients, associated with increased comorbidities and prolonged hospitalization [56]. Another retrospective study conducted in two hospitals in Jeddah, Saudi Arabia, found a 5 % annual rise in CRE prevalence, with most isolates susceptible to tigecycline and colistin [57].

### 3.4 Multidrug-Resistance (MDR)

The WHO categorizes MDR bacteria into critical, high, and medium priority groups, with the most critical group (including *Acinetobacter*, *Pseudomonas,* and various Enterobacteriaceae (including *Klebsiella,* *Escherichia coli*, *Serratia*, and *Proteus*)) posing a significant threat in hospitals, nursing homes, and patients requiring ventilators and blood catheters [58]. MDR bacteria are categorized into three groups based on their resistance profiles: a) Multi-Drug Resistant Organisms (MDROs), which are non-susceptible to at least one agent in three or more antimicrobial categories; b) Extensively Drug-Resistant organisms (XDRs), which are non-susceptible to at least one agent in all but two or fewer antimicrobial categories; and c) Pan-Drug-Resistant organisms (PDRs), which are non-susceptible to all agents. A new recommendation has been launched to classify infections caused by Gram-positive and emerging Gram-negative multidrug-resistant pathogens: *Enterococcus faecium*, *Staphylococcus aureus*, *Klebsiella pneumoniae*, *Acinetobacter baumannii*, *Pseudomonas aeruginosa*, and *Enterobacter* spp. (ESKAPE), which are responsible for over 80% of infectious episodes in the ICU [59].

A study conducted in Riyadh, Saudi Arabia, found that 60% of Enterobacteriaceae cultures were MDR or XDR infections, with *Escherichia coli* being the most common, followed by *Klebsiella pneumoniae* and *Enterobacter sp*. Piperacillin/tazobactam (53%), carbapenems (47%), and cephalosporins (21.3%) were the most preferred antibiotics. The case fatality rate (84%) was significantly higher in 138 patients with MDR and XDR Enterobacteriaceae, with ventilator use and ICU stays exceeding 72 hours, causing increased hospital stays [60].

*Acinetobacter baumannii*, an opportunistic Gram-negative bacterium, is a common cause of healthcare-associated infections, especially in ICUs. A study reported the prevalence of multidrug-resistant *Acinetobacter baumannii* in the Aseer region of the Kingdom of Saudi Arabia. In this study, the antibiotic susceptibility of ninety-four (n = 94) clinical isolates of *A. baumannii* collected over a period of 3 months in the last quarter of 2015 was assessed. The isolates were resistant to several antibiotics, including carbapenems and aminoglycosides, making treatment difficult. It is, therefore, considered a new threat in hospitals that requires enormous efforts to stop its escalation and spread [61].

*Pseudomonas aeruginosa* is also an opportunistic pathogen that can cause severe infections in ICU patients, including ventilator-associated pneumonia and bloodstream infections. A study encompassed all *P. aeruginosa* infections that emerged more than 48 hours after hospital admission within the period from January 31, 2016, to December 31, 2018, at ICUs affiliated with King Abdulaziz Medical City in Riyadh, Saudi Arabia. MDR strains of *P. aeruginosa* limit treatment options and increase the risk of treatment. Many *P. aeruginosa* isolates were from patients suffering from respiratory tract and wound infections. The highest antibiotic resistance was found with meropenem, followed by ticarcillin, imipenem, and piperacillin. About 13% of strains were multidrug resistant. Moreover, the study identified several potentially modifiable factors associated with *P. aeruginosa* infection in ICUs [62]. A recent study at King Abdullah Hospital in Bisha, Saudi Arabia, found *Klebsiella pneumoniae* MDR infection in 66.8% of adult patients, highlighting the prevalence and risk factors associated with this infection [63]. The resistance rate of MDR *Klebsiella pneumoniae* was highest for ampicillin (100%), cefuroxime (97.9%), ceftriaxone (94.3%), and aztreonam (92.2%), while lowest for colistin (16.3%) and tigecycline (6.4%). Furthermore, MDR *Klebsiella pneumoniae* infection was significantly associated with patients' gender, age group, ICU admission, invasive medical devices, and chronic illness, with male gender, patients over 65, and ICU admission being independent risk factors.

### 3.5 Vancomycin-Resistant Enterococci (VRE)

Vancomycin-resistant strains of enterococci can cause infections in ICU patients, especially those with indwelling catheters or prolonged hospital stays. VRE infections are associated with increased morbidity and mortality rates and require alternative treatment options. A study conducted at King Khalid University Hospital, a tertiary care hospital in Riyadh, Saudi Arabia, in 2015 focused on inpatients diagnosed with enterococci infection. The study identified 231 enterococci isolates from blood, urine, and body fluids, with 82.7% being Vancomycin-Susceptible Enterococci (VSE) and 17.3% being VRE. Notably, VRE was more common in blood samples, whereas VSE was more prevalent in urine samples. The study revealed a high prevalence of VRE in ICUs and high-risk areas, such as oncology units, emphasizing the need for further investigation to enhance infection management [64]. A retrospective study at King Fahad Specialist Hospital in Dammam, conducted on 30 ICU patients, found that prior antibiotic use was a significant factor in VRE cases. Furthermore, patients admitted to high-risk ICU rooms were protected, indicating the importance of strict isolation precautions in high-risk ICU rooms [41].

## 4 MOLECULAR PATTERNS OF BACTERIAL RESISTANCE

Bacterial resistance, a significant public health issue, is particularly prevalent in the ICU, where immunocompromised patients receive multiple antibiotic courses due to their intrinsic and acquired resistance mechanisms. Intrinsic resistance mechanisms are innate to bacterial species and are not due to acquired mutations or genetic alterations. In contrast, acquired resistance mechanisms result from genetic mutations or the acquisition of resistance genes from other bacteria. The genomic patterns of bacterial resistance are complex and multifactorial, reflecting the multiple processes by which bacteria can acquire and maintain resistance to antibiotics [65]. A common resistance mechanism is the acquisition of plasmids or other mobile genetic elements that carry resistance genes. These plasmids can spread between bacterial cells through conjugation, transformation, or transduction, rapidly disseminating the resistance genes within bacterial populations [66, 67]. Another mechanism of resistance is a mutation of chromosomal genes, which can lead to changes in bacterial metabolism or in the structure or function of bacterial proteins targeted by antibiotics. These mutations may occur spontaneously or be triggered by exposure to antibiotics. They can occur in genes involved in various aspects of bacterial physiology, including cell wall synthesis, DNA replication, and protein synthesis. In addition, bacteria can develop resistance through changes in regulatory networks that control gene expression. For example, bacteria can upregulate efflux pumps that remove antibiotics from the cell or downregulate membrane transporters that allow antibiotics to enter the cell. Biofilms are bacterial communities that attach to surfaces and are enclosed in a matrix of Extracellular Polymeric Substances (EPS). Bacteria in biofilms are highly resistant to antibiotics due to the protective nature of the EPS matrix. Biofilms are commonly found in medical devices such as catheters and endotracheal tubes, making them an important source of infection in the ICU [68].

Beta-lactam antibiotics, such as penicillin and cephalosporins, are commonly used in ICUs; however, many bacteria produce beta-lactamases, enzymes that break down beta-lactam antibiotics, rendering them ineffective. The production of beta-lactamases is an effective mechanism of bacterial resistance in the ICU. A 2018 study published in the Saudi Medical Journal reported a high prevalence of resistance to cephalosporins in bacteria isolated from ICU patients in Saudi Arabia [69]. The clinical importance of *Pseudomonas aeruginosa* has increased significantly due to its ability to rapidly develop resistance to various groups of antibiotics. *Pseudomonas aeruginosa* and *Acinetobacter baumannii* are exhibiting resistance against antibiotic therapies due to efflux pumps of bacterial membrane transporters. Among patients in the ICU of the military hospital in Riyadh, Saudi Arabia, *Acinetobacter baumannii* was the most frequently isolated bacterium in the ICU, accounting for 40.9% of the samples. *Acinetobacter* was the leading cause of ventilator-associated pneumonia at King Abdulaziz Medical City in Riyadh from 2003 to 2009, with bacterial resistance increasing from 19% in 2006 to 81.5% in 2015 and 92.1% in another study [70-72]. It has been reported that prolonged intubation in intensive care patients can cause ventilator-associated pneumonia due to the formation of a bacterial biofilm on the surface of the equipment [73]. Another study involving three hospitals in Riyadh, Saudi Arabia, analyzed 25 clinical isolates of *Acinetobacter baumannii* using whole-genome sequencing to understand the genetic determinants of antibiotic resistance. The results revealed several resistance genes in the *Acinetobacter baumannii* isolates, including genes encoding beta-lactamases, aminoglycoside-modifying enzymes, and efflux pumps. It's important to note that some bacteria may have multiple resistance genes, and resistance can also arise through other mechanisms, such as changes in membrane permeability or efflux pumps. The major bacterial resistance genes responsible for antibiotic resistance in ICU settings are:

### 4.1 mecA

The mecA gene is a gene that codes for a protein that makes bacteria resistant to methicillin and other related antibiotics. The mecA gene, linked to bacterial resistance, is a significant genetic factor in MRSA strains, complicating treatment options and limiting the effectiveness of commonly used antibiotics. This may lead to an increase in morbidity, mortality, and healthcare costs associated with MRSA infections. A previous study determined the prevalence of *Staphylococcus epidermidis* as well as the pattern of antibiotic susceptibility and mecA prevalence in *Staphylococcus epidermidis* isolated from patients in ICUs [74-76]. The emergence of mecA-mediated resistance to β-lactam antibiotics can be largely attributed to the extensive human use of these agents, not only for treating infections in humans but also as growth promoters in animal feed. This resistance evolution was driven by a series of critical genetic events: (1) transformation of a native gene into a functional resistance determinant at its original genomic site, (2) independent evolution of Staphylococcal Cassette Chromosome (SCC) elements, particularly at the orfX integration site, (3) incorporation of the mec gene complex and adjacent regions into the SCC element, giving rise to SCCmec, (4) subsequent adaptation of the host bacterial genetic background to accommodate and maintain the resistance element, (5) horizontal dissemination of SCCmec among Staphylococcus species colonizing animals, and (6) eventual spread of SCCmec among strains colonizing both animals and humans. Notably, many of these evolutionary steps appear to have occurred within primitive, animal-associated *Staphylococcus* species, often isolated from livestock or zoonotic infections, highlighting a bacterial survival mechanism in response to anthropogenic antibiotic pressure. The eventual cross-species transfer of SCCmec into human-adapted pathogens, particularly *Staphylococcus aureus*, represents a pivotal event that catalyzed the emergence of globally significant, β-lactam-resistant strains and multiple pandemic waves of MRSA [77].

### 4.2 blaCTX-M Gene

The blaCTX-M gene encodes a type of beta-lactamase enzyme responsible for antibiotic resistance in bacterial organisms. It is one of the most common ESBL types and is frequently found in *Escherichia coli* and other Gram-negative bacteria. BlaCTX-M type ESBLs, the most common ESBLs in Saudi Arabia, pose a significant challenge in treating bacterial infections due to their limited antibiotic efficacy [78]. Globally, the detection rate of ESBL-positive *Escherichia coli* in ICUs varies, ranging from 12.8% in Canada to 70.6% in Iran [79]. Almost all the ESBL-*Escherichia coli* isolates in the Riyadh region of Saudi Arabia were found to be resistant to cephalosporins and penicillin [80].

Recently, a study revealed that blaCTX-M-positive ESBL-containing *Escherichia coli* and *Klebsiella pneumoniae* isolates originated from an outbreak in a Neonatal Intensive Care Unit (NICU) in the Ha'il region of Saudi Arabia [78]. A previous study documented the high prevalence of ESBLs in *Escherichia coli* isolates in tertiary hospitals in Riyadh, where blaCTX-M-15 was the predominant ESBL gene [81].

The *Escherichia coli* isolates demonstrated varying degrees of resistance to ESBL antibiotics, with particularly high resistance rates observed for ampicillin (96.61%), aztreonam (89.83%), ceftazidime (81.36%), ciprofloxacin (79.66%), cefotaxime (76.27%), and cefepime (75.58%). Resistance to cefoxitin was comparatively lower (15.25%), while no resistance was detected against carbapenems such as meropenem and imipenem. These resistance patterns were corroborated by oligonucleotide microarray-based assays and Polymerase Chain Reaction (PCR) genotyping, which confirmed the presence of key β-lactam resistance genes. Among the isolates, sequence type ST131 emerged as the predominant lineage, recognized globally for its heightened uropathogenicity and MDR. Additionally, the isolates harbored genes conferring resistance to aminoglycosides, further complicating therapeutic options. The rapid emergence and dissemination of ESBL-producing *Escherichia coli* in Saudi Arabia may be exacerbated by the country's high seasonal influx of visitors, particularly to the city of Makkah. This raises significant public health concerns regarding the potential for widespread transmission and therapeutic failure. In light of these findings, there is an urgent need for robust antimicrobial resistance surveillance systems across general hospitals in Saudi Arabia. Proactive monitoring and antimicrobial stewardship interventions will be critical in curbing the escalating trend of drug-resistant infections and preserving the efficacy of current treatment regimens [82].

### 4.3 blaOXA-23 Gene

The blaOXA-23 gene is a Carbapenem-Hydrolyzing class D β-lactamase (CHDL) gene first identified in a clinical isolate of *Acinetobacter baumannii*. Expression of blaOXA-23 by *Acinetobacter baumannii* poses a significant clinical issue due to its resistance to beta-lactam antibiotics, including carbapenems, which are often the last resort for multidrug-resistant infections. The spread of blaOXA-23 in the ICU can occur through several mechanisms, including horizontal gene transfer, in which resistance genes are exchanged between bacteria, and clonal expansion, in which a particular strain of bacteria carrying the gene becomes dominant. These mechanisms contribute to the widespread of carbapenem-resistant *Acinetobacter baumannii* strains in ICUs, resulting in increased morbidity and mortality. The presence of blaOXA-23-mediated resistance in Saudi Arabian ICUs poses a major challenge for patient management and infection control [83-85].

### 4.4 blaOXA-66 Gene

The blaOXA-66 gene is a beta-lactamase gene that is abundant in certain bacteria, particularly in the Enterobacteriaceae family. This gene encodes a specific blaOXA-66-type beta-lactamase variant that confers resistance to a wide range of beta-lactam antibiotics. In recent years, the emergence and spread of Carbapenem-resistant bacteria, including those carrying the blaOXA-66 gene, have posed a significant threat to patient health in ICUs worldwide, including Saudi Arabia, due to limited treatment options and increased mortality rates [86]. The prevalence of blaOXA-66 and other carbapenemase genes in Saudi ICUs may vary depending on the region and specific healthcare facility. However, several studies have reported the presence of carbapenem-resistant bacteria and corresponding resistance genes in Saudi Arabian hospitals, including ICUs [87].

A cross-sectional study conducted between September 2017 and August 2018 at King Abdullah Hospital in Bisha, Saudi Arabia, highlighted the genetic diversity of ESBL determinants circulating among Gram-negative bacteria in a regional healthcare setting. The predominance of *blaTEM* and the frequent presence of multiple ESBL genes within single isolates signal a pressing need for expanded molecular surveillance. Implementing routine molecular diagnostics at hospital laboratories could strengthen infection control strategies and guide more effective empirical antibiotic therapy. National-level surveillance programs are also essential to map the transmission dynamics and inform antimicrobial stewardship policies across healthcare institutions in Saudi Arabia [88].

## 5 STRATEGIES OF THE WHO AND SAUDI ARABIA ON ANTIBIOTIC RESISTANCE IN THE ICU

The WHO has recognized antibiotic resistance as a global health problem and has identified measures to combat the problem in various healthcare settings, including ICUs. The increasing emergence and spread of MDR bacteria in Saudi Arabia are attributed to the unoptimized use of antibiotics, the sale of over-the-counter antibiotics without prescription, a high volume of international travel (resulting in a significant number of expatriates and their pilgrimages to holy cities), and challenges in adhering to infection control practices [89]. Saudi Arabia's Ministry of Health introduced antimicrobial containment and infection prevention programs in hospitals in 2015 and developed its first AMR National Action Plan in 2017 [90]. The first AMR National Action Plan adopted the five objectives of the WHO Global Action Plan on AMR, aiming to improve awareness, strengthen knowledge, reduce the incidence of infections, optimize the use of antimicrobial medicines, and develop an economic case for sustainable investment in new medicines, diagnostic tools, vaccines, and other interventions [91].

Many of the specific strategic objectives outlined in the 2017 National Action Plan have been implemented effectively, while others are still in various phases of development [90]. The revised plan is an updated supplement to the National Strategy for Combating Antimicrobial Resistance (2022–2025), developed in collaboration with multisectoral stakeholders and detailing the Kingdom's efforts to combat AMR, its development process, and clear actions for the period. Furthermore, the National AMR Committee, formed in 2017, consists of scientists and experts from various sectors and was restructured in 2019. It supported the development of a 2022 strategy and action plan.

The following are some of the key strategies that the National AMR Committee, in partnership with WHO, uses to address antibiotic resistance in intensive care units:

### 5.1 Surveillance and Monitoring

Surveillance and monitoring of antibiotic resistance in ICUs is critical for infection prevention and control of drug-resistant pathogens. The WHO emphasizes the importance of surveillance systems in monitoring antibiotic resistance trends in ICUs, which involve collecting data on antibiotic use, resistance rates, and the spread of resistant bacteria. The data collected helps in decision-making and action [91, 92]. The WHO has also been supported by Saudi Arabia since 2015 and has provided over $385 million to global health initiatives and emergency relief efforts in various countries, including Bangladesh, Iraq, Somalia, Syria, and Yemen [91].

Saudi Arabia joined the WHO Global Antimicrobial Resistance Surveillance System (GLASS) in May 2017, identifying eight pathogens (*Escherichia coli*, *Klebsiella pneumoniae*, *Staphylococcus aureus*, *S. pneumoniae*, *Salmonella* spp., *Shigella* spp., and *N. gonorrhoeae*) for early implementation [93]. The national plan endorsed the GLASS protocol, and 42 laboratories are available nationwide for surveillance and testing for antibiotic susceptibility [90]. National AMR surveillance studies can help identify the problem by collecting bacterial isolates from various sites, testing their susceptibility to antibiotics, and determining the Minimum Inhibitory Concentration (MIC). A study conducted by Thabit *et al.* evaluated the last decade's (2013–2023) AMR surveillance in Saudi Arabia, revealing that most studies were either single-center, limited to specific bacterial species, or did not use Broth Microdilution (BMD) as the testing method [94]. The study also emphasized the need for large-scale, globally recognized surveillance studies.

### 5.2 Infection Prevention and Control (IPC)

The committee recommends that ICUs implement strict IPC measures to prevent healthcare-associated infections and reduce antibiotic use. These include hand hygiene, proper use of personal protective equipment, cleaning of the environment, and adherence to standard precautions. IPC affects every aspect of healthcare, including hand hygiene, surgical site infections, injection safety, antibiotic resistance, and non-emergency hospital operations. The WHO has adopted a 'Global Strategy for Infection Prevention and Control' through a resolution that seeks to position IPC as central to infectious hazards and responses to infection threats and public health emergencies and essential to addressing the silent burden of HAIs and AMR [95].

Saudi Arabia follows WHO infection prevention and control policies, implementing screening schemes for high-risk patients and isolation precautions to limit the spread of MDR pathogens. Healthcare workers in Saudi hospitals possessed excellent knowledge of risk management plans and pandemic sub-plans related to infection control and prevention policies, procedures, and principles, which has aided the health authorities in Saudi Arabia in mitigating COVID-19 effectively [96].

### 5.3 Rational Antibiotic Use

The WHO advocates for the appropriate and judicious use of antibiotics in ICUs. This includes implementing antimicrobial stewardship programs to optimize prescribing practices, ensure accurate diagnosis and treatment, and prevent misuse or overuse of antibiotics. The WHO has worked with the Saudi MOH to promote the rational use of antibiotics in Saudi Arabia [91]. A recent study conducted in Saudi Arabia aimed to assess the population's knowledge, attitudes, and practices (KAP) regarding the use of antibiotics. The study found that, despite new legal regulations on antibiotic dispensing, misuse of antibiotics remains evident, underscoring the need for awareness campaigns and educational programs to enhance antibiotic knowledge [97, 98].

The Saudi MOH implemented a nationwide antibiotic restriction policy in April 2018, imposing penalties such as license revocation, fines, and imprisonment of up to 6 months for non-compliance [99]. A study conducted after the MOH restriction policy went into effect found that, despite the policy's intended limit on over-the-counter antibiotic use, there was a significant increase in parenteral antibiotic use. The study attributed inappropriate prescribing practices as one of the factors contributing to this increase [100].

### 5.4 Education and Training

WHO emphasizes the significance of education and training for healthcare professionals working in ICUs on its website [101]. This includes promoting awareness of antibiotic resistance, training on IPC measures and rational antibiotic use, and disseminating guidelines and best practices. In Saudi Arabia, healthcare workers receive training and education on antibiotic use as part of their medical and nursing education. The Saudi MOH, along with various medical universities and institutions, plays an important role in providing training and guidelines to health professionals on the appropriate use of antibiotics [97]. The Saudi MOH has developed national guidelines and protocols for the appropriate use of antibiotics. These guidelines describe the indications, dosages, dwell times, and prescribing practices for various antibiotics. Healthcare workers are encouraged to follow these guidelines to promote the rational use of antibiotics.

Antibiotic Stewardship Programs (ASP) are crucial for optimizing antibiotic use in healthcare facilities, [102] with many Saudi Arabian facilities implementing these programs to promote responsible antibiotic use. These programs involve multidisciplinary teams working together to develop protocols, monitor antibiotic use, train healthcare professionals, and implement measures to optimize antibiotic prescribing practices [103]. It aims to control the development and transmission of Multidrug-Resistant Organisms (MDROs) through appropriate prescribing, early detection and prevention, and reservoir elimination. It's essential to note that, despite efforts to educate and train healthcare professionals on the appropriate use of antibiotics, in some cases, adherence to guidelines remains a problem, leading to the overuse of antibiotics. However, ongoing education and training initiatives are designed to help overcome these challenges and improve the overall understanding and practice of antibiotic use among healthcare workers in Saudi Arabia.

A study in a tertiary care hospital in Riyadh found that implementing ASP improved the prescribing appropriateness rate of empirical antibiotic therapy to 100% [104]. The ASP group initially used 79.1% of antibiotics inappropriately; however, this rate was reduced to 0% after implementing the recommendations. Nonetheless, the implementation of ASP reduced antibiotic utilization and direct costs. Another study conducted in Makkah province demonstrated that ASP led to a significant decrease in the use of restricted antimicrobials, such as piperacillin-tazobactam (66.5%), ceftriaxone (82.3%), and vancomycin (66%) [105]. However, it increased the usage of antibiotics like meropenem (54%), linezolid (100%), and levofloxacin (66%) due to their timely delivery in life-threatening infectious diseases.

## 6 PREVENTION OF ANTIMICROBIAL RESISTANCE AND ITS CHALLENGES

Antibiotics have undeniable benefits in treating bacterial infections, but their overuse and improper use can lead to antibiotic resistance and other problems. Implementing preventive measures, promoting responsible antibiotic use, and exploring alternative treatments are critical to addressing these challenges and preserving the effectiveness of antibiotics for future generations. One of the most important prevention strategies is raising public awareness about the appropriate use of antibiotics. Educating the public about the difference between viral and bacterial infections can help reduce unnecessary antibiotic usage and prevent misconceptions about antibiotic effectiveness [102]. Awareness campaigns can be conducted to talk about the safe use of antibiotics. An online quiz game comprising questions on AMR knowledge and antibiotic handling was conducted to assess the knowledge and raise awareness about AMR. This study showed that social media played a major role as a source of health information and recommended the use of reliable and good-quality videos from YouTube to promote AMR awareness [106].

The Kingdom plans to measure AMR reduction using indicators related to five pathogens and antimicrobial agent use. The outcome indicators include the rate of *Escherichia coli* in blood specimens resistant to 3rd generation cephalosporins, methicillin-resistant *Staphylococcus aureus*, carbapenem-resistant *Klebsiella pneumoniae/Escherichia coli*/*Pseudomonas aeruginosa*, and colistin-resistant *Acinetobacte*r, and the rate of a defined daily dose of selected antimicrobials (Meropenem, Imipenem, Piperacillin /tazobactam, Colistin, and Vancomycin) per 100 patient-days [90]. The current AMR action plan predicts that these indicators will become more significant as hospital AMR reporting improves. The national committee plans to actively monitor these indicators, to begin reporting by 2024.

Health professionals play an important role in preventing AMR by prescribing antibiotics wisely. They should follow evidence-based guidelines and prescribe antibiotics only when needed. This includes proper diagnosis, considering alternative treatment options, and selecting the appropriate antibiotic at the correct dose and duration. Furthermore, stewardship programs help prevent the emergence and spread of antibiotic resistance by ensuring the proper use of antibiotics. Poor adherence to ASP regulations has resulted in limited adoption of antimicrobial stewardship strategies in Saudi MOH hospitals, despite a national policy for their implementation [107]. In fact, the government of Saudi Arabia has implemented a law to prevent the dispensing of unprescribed antibiotics. However, a survey conducted in 2023 assessing the impact of this law concluded that not all physicians agree that it will prevent illegal dispensing. This indicates that enforcing laws is not sufficient on its own [108]. Furthermore, inadequate communication among key stakeholders, a shortage of ASP team members, and a lack of awareness among hospital management all impede the successful implementation of ASP in Saudi hospitals. Hence, pharmacists and the pharmacy department must coordinate with physicians and nurses for better antimicrobial stewardship initiatives. Additionally, physicians in Saudi hospitals face challenges in adopting ASP due to liability pressure, fear of patient deterioration, and concerns about the risks and benefits of ASP for current patients, not future ones.

AMR is a global problem that requires international cooperation and coordination. Harmonization of regulations, sharing of surveillance data, and implementation of strategies across countries are critical to effectively addressing antibiotic resistance [109]. Notably, the average prevalence of MRSA among all *Staphylococcus aureus* isolates in studies from Saudi Arabia was approximately 32.5%, which is higher than global averages reported in various settings. For context, the global all-age mortality rate attributable to AMR decreased from 19.8 deaths per 100,000 in 1990 to 14.5 per 100,000 in 2021, but Saudi-specific data highlight a higher burden of resistant organisms such as MRSA within ICU settings. These comparisons emphasize the need for stronger, context-specific infection control and antimicrobial stewardship strategies in Saudi Arabia [94, 110]. The discovery and development of new antibiotics is essential to combating antibiotic resistance. Encouraging research, providing incentives, and promoting collaboration among academia, industry, and government agencies can help stimulate the discovery of new antibiotics. Antibiotic misuse and overuse significantly contribute to AMR, requiring education and strict adherence to guidelines to prevent misuse, improper prescribing, and self-medication. Antibiotic use in agriculture, especially in livestock, contributes significantly to AMR. Antibiotics are sometimes used as growth promoters or to prevent disease in animals. Resistant bacteria from animals can be transmitted to humans through the food chain and the environment (*via* animal waste), thereby increasing the risk of antibiotic resistance. Implementing regulations and promoting responsible use of antibiotics in agriculture are necessary to address this challenge [111].

Developing new antibiotics poses a significant financial challenge due to the high costs and low returns on investment. Pharmaceutical companies often struggle to recoup the costs of research and development. Economic incentives, such as market guarantees and subsidies, can help encourage investment in antibiotic research [112]. The limited availability of treatment alternatives to antibiotics is a challenge, especially in cases where antibiotics are necessary. Research and development of alternative therapies, such as phage therapy and novel antimicrobial agents, can help address this problem. Addressing the challenges and implementing effective prevention strategies are critical to maintaining antibiotic efficacy and combating AMR. This requires a multifaceted approach in which health professionals, policymakers, researchers, and the public work together to ensure the responsible use of antibiotics.

## CONCLUSION

Antibiotic resistance is a major problem in healthcare, especially in ICUs. In Saudi Arabia, the emergence and spread of antibiotic-resistant bacteria pose a major challenge to patient care and clinical management. After reviewing the available data, several conclusions can be drawn regarding the clinical impact and patient outcomes associated with antibiotic resistance in the Saudi ICU. Antibiotic resistance increases the risk of healthcare-associated infections. The spread of MDROs within healthcare facilities can lead to outbreaks and transmission of resistant strains to vulnerable patients. Continuous surveillance of antibiotic resistance patterns and ongoing research efforts are needed to monitor the prevalence and trends of resistance in the Saudi ICU. This approach should include strict adherence to infection control measures, implementation of antibiotic resistance programs, continuous surveillance, and ongoing research efforts to address the issue. Promoting prudent antibiotic use and addressing the spread of resistant bacteria can mitigate the impact of antibiotic resistance on patient care in the Saudi ICU. While numerous strategies are being implemented in Saudi Arabia to control antibiotic resistance, the need for public awareness campaigns, prescription regulations, and ASPs remains. Well-structured patient follow-up protocols are crucial for monitoring compliance and assessing therapeutic response. Additionally, efforts must be made to refine existing strategies and develop new ones to ensure that antibiotic resistance is effectively controlled. Mandatory training on ASP, proper implementation, continuous and strict monitoring, allocation of the required resources, and interdisciplinary collaboration may help in controlling antibiotic resistance. Upon strategic implementation, these measures may also be effective in low-resource settings where cost-effective interventions are valuable. Future research in a diverse population, including those in rural areas and of different age groups, with a focus on a specific treatment-resistant organism/gene, may be beneficial to assess the impact of AMR and for the development and implementation of effective strategies. Focusing on evidence-based outcomes for patient-centered interventions may help in combating antibiotic resistance on a national level.

## Figures and Tables

**Fig. (1):**
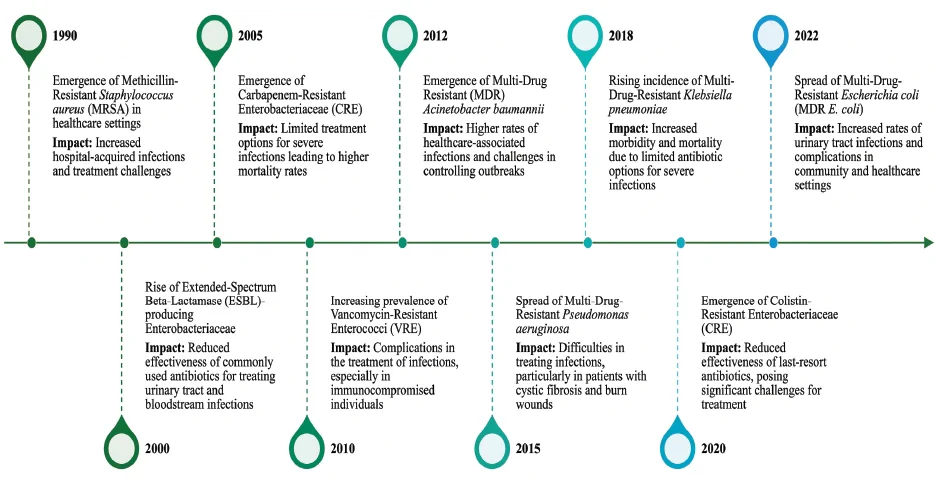
Emergence and impact of bacterial resistance in Saudi Arabia: A timeline analysis [37-46].

**Table 1:** Hospital-Acquired Infections (HAIs) in Saudi Arabia: Concerns and implications in intensive care units (ICUs).

**HAIs in Saudi Arabia, Particularly in ICUs**	**Overview**	**References**
Prevalence	HAIs are a major problem in Saudi Arabia, especially in ICUs. HAIs pose a considerable burden on patients, healthcare providers, and healthcare systems.	[27]
Common Types	The most common types of HAIs in Saudi Arabian ICUs include ventilator-associated pneumonia (VAP), bloodstream infections (BSIs), urinary tract infections (UTIs), and surgical site infections (SSIs).	[28]
Contributing Factors	Several factors contribute to the prevalence of HAIs in Saudi Arabian ICUs, including poor hand hygiene, inadequate infection control measures, improper sterilization practices, overcrowding, and antibiotic resistance.	[29]
Impact on Patients	HAIs can significantly impact patients in ICUs, prolonging their hospital stay, increasing morbidity and mortality rates, and leading to additional healthcare costs. The vulnerable nature of ICU patients, compromised immune systems, and invasive procedures make them particularly susceptible to HAIs.	[30]
Impact on Healthcare Providers and Systems	HAIs pose a substantial burden on healthcare providers and systems in Saudi Arabia. They increase the workload of healthcare staff and may result in a shortage of ICU beds for critically ill patients. Additionally, HAIs can negatively affect the reputation of healthcare facilities and their ability to provide quality care.	[31]
Prevention and Control Measures	Saudi Arabia has implemented various strategies to prevent and control HAIs, including promoting hand hygiene practices, implementing strict infection control protocols, educating healthcare providers about proper sterilization techniques, and promoting antimicrobial stewardship programs to combat antibiotic resistance. Continuous monitoring and surveillance of HAIs are also crucial to identify trends, implement interventions, and improve patient safety.	[32]
Future Initiatives	The Saudi Arabian government and healthcare authorities are continuously working on improving infection control practices and reducing the burden of HAIs. They are investing in training programs for healthcare workers, enhancing surveillance systems, and implementing advanced technologies for infection prevention and control. Collaboration between healthcare facilities and regulatory bodies is encouraged to ensure adherence to guidelines and best practices.	[26]

## LIST OF ABBREVIATIONS

AMR: Antimicrobial Resistance
ASP: Antibiotic Stewardship Programs
BMD: Broth Microdilution
BSI: Bloodstream Infections
CHDL: Carbapenem-Hydrolyzing Class D β-Lactamase
CRE: Carbapenem-Resistant Enterobacteriaceae
EPS: Extracellular Polymeric Substances
ESBL: Extended-Spectrum Beta-Lactamase
ESKAPE: *Enterococcus faecium, Staphylococcus aureus, Klebsiella pneumoniae, Acinetobacter baumannii, Pseudomonas aeruginosa,* and *Enterobacter* spp.
GLASS: Global Antimicrobial Resistance Surveillance System
GNB: Gram-Negative Bacilli
HAIs: Hospital-Acquired Infections
ICUs: Intensive Care Units
IPC: Infection Prevention and Control
KAP: Knowledge, Attitudes, and Practices
MAR: Multiple Antibiotic Resistance
MDR: Multi-Drug Resistant
MDROs: Multi-Drug Resistant Organisms
MOH: Ministry of Health
MRSA: Methicillin-Resistant *Staphylococcus aureus*
NICU: Neonatal Intensive Care Unit
PCR: Polymerase Chain Reaction
PDRs: Pan-Drug-Resistant Organisms
SSIs: Surgical Site Infections
UTIs: Urinary Tract Infections
VAP: Ventilator-Associated Pneumonia
VRE: Vancomycin-Resistant Enterococci
VSE: Vancomycin-Susceptible Enterococci
WHO: World Health Organization
XDRs: Extensively Drug-Resistant Organisms
